# Definition of aspirin resistance using whole blood impedance aggregometry in patients undergoing coronary artery surgery: methodological challenges and outcome improvement opportunities

**DOI:** 10.1186/1749-8090-8-S1-P122

**Published:** 2013-09-11

**Authors:** M Petricevic, B Biocina, S Konosic, I Burcar, V Ivancan, D Strapajevic, L Svetina, R Habekovic, Z Hizar, H Gasparovic

**Affiliations:** 1 Department of Cardiac Surgery ; University Hospital Center Zagreb, Zagreb, Croatia; 2 Department of Cardiac Anesthesiology ; University Hospital Center Zagreb, Zagreb, Croatia

## Background

A beneficial effect of acetylsalicylic acid (ASA) on vein graft patency has been described, but some patients experience adverse cardiac events despite appropriate ASA treatment. Study aim was to define ASA resistance using Multiple electrode aggregometry (MEA) preoperatively in group of patients undergoing coronary artery bypass grafting (CABG).

## Methods

Prospective observational trial at University Hospital Center Zagreb enrolled 131 patients scheduled for CABG, and divided them into 4 groups with respect to preoperative antiplatelet therapy (APT). Group 1 received 100 mg ASA per day, Group 2 100 mg ASA + 75 mg clopidogrel per day, Group 3 75 mg clopidogrel per day, and Group 4 did not receive any APT. MEA with ASPI test (sensitive to ASA) and ADP test (sensitive to clopidogrel) was performed prior to surgery. In Group 1, patients were characterized as ASA resistant if their ASPI test value exceeded the 75th percentile distribution.

## Results

Study enrolled 131 patients. Significant differences both in the ASPI (p<0.001) and the ADP test (p=0.038) were observed between patients in different APT groups. In Group (1) ASPI test value of 30 AUC presented 75th percentile of distribution, thus indicating ASA resistance. Group 2 patients had slightly lower ADP test values, but no significant difference occurred (mean 60.05 vs. 63.32 AUC, p=0.469). In Group 1 and 2, significant correlations between the ADP test and both, platelet count (r=0.347, p<0.001) and fibrinogen level (r=0.364, p<0.001) were observed.

## Conclusion

Association between low response to ASA and post-CABG major adverse ischemic events risk increase has been described thus indicating need for ASA resistant patients detection using bedside suitable and drug specific platelet function tests. In patients with preoperative ASPI test exceeding 30 AUC postoperative, ASA dose adjustment or clopidogrel addition according to MEA results should be considered.

